# Function of Adenylyl Cyclase in Heart: the AKAP Connection

**DOI:** 10.3390/jcdd5010002

**Published:** 2018-01-16

**Authors:** Tanya A. Baldwin, Carmen W. Dessauer

**Affiliations:** Department of Integrative Biology and Pharmacology, McGovern Medical School, University of Texas Health Science Center, Houston, TX 77030, USA; Tanya.Baldwin@uth.tmc.edu

**Keywords:** adenylyl cyclase, A-kinase anchoring proteins, cyclic AMP, cardiomyocytes

## Abstract

Cyclic adenosine monophosphate (cAMP), synthesized by adenylyl cyclase (AC), is a universal second messenger that regulates various aspects of cardiac physiology from contraction rate to the initiation of cardioprotective stress response pathways. Local pools of cAMP are maintained by macromolecular complexes formed by A-kinase anchoring proteins (AKAPs). AKAPs facilitate control by bringing together regulators of the cAMP pathway including G-protein-coupled receptors, ACs, and downstream effectors of cAMP to finely tune signaling. This review will summarize the distinct roles of AC isoforms in cardiac function and how interactions with AKAPs facilitate AC function, highlighting newly appreciated roles for lesser abundant AC isoforms.

## 1. Introduction

The heart continuously balances the interplay of various signaling mechanisms in order to maintain homeostasis and respond to stress. One pathway that contributes to cardiac physiology and stress is the cyclic adenosine monophosphate (cAMP) pathway. cAMP is a universal second messenger that integrates input from G-protein-coupled receptors to coordinate subsequent intracellular signaling. Synthesis of cAMP from adenosine triphosphate (ATP) is controlled by the enzyme adenylyl cyclase (AC). In the heart, cAMP acts downstream on a variety of effectors including protein kinase A (PKA), hyperpolarization-activated cyclic nucleotide-gated channels (HCN), exchange protein directly activated by cAMP (EPAC), Popdc proteins, and a fraction of phosphodiesterases (PDEs). PKA is the most well-known and studied cAMP effector. PKA phosphorylation of intracellular targets coordinates a number of physiological outputs including contraction [1,2] and relaxation [3]. HCN channel regulation by cAMP maintains basal heart rate [4] while EPAC facilitates calcium handling and cardiac hypertrophy [5]. PDEs degrade cAMP, further defining the temporal regulation of the signal. The most recently discovered cAMP effector, Popdc, is important for heart rate dynamics through regulation of the potassium channel TREK1 [6]. 

The AC family is composed of nine membrane-bound isoforms (AC 1–9) and one soluble isoform (sAC). All of the isoforms can be found in the heart with the exception of AC8 [7,8]. Cardiac fibroblasts express AC 2–7 [9], while in adult cardiac myocytes AC5 and AC6 are considered the major isoforms [10,11]. Lower levels of AC2, AC4, and AC9 are reported in myocytes [12,13].

## 2. Adenylyl Cyclases (ACs) and Their Role in Cardiac Function: Knockout Phenotypes

AC5 and AC6 are closely related isoforms that share similar regulatory mechanisms, including inhibition by Gαi as the hallmark of this group; however, physiologically they appear to play distinct roles in cardiac function [8,14]. Additional modes of regulation for AC5/6 are extensively reviewed elsewhere [15]. AC5 and AC6 are differentially expressed in development, with age, and in a pressure overload model of cardiac hypertrophy where an increase in AC5 protein is observed in neonatal heart and models of heart disease [16,17]. Another potential distinction between these two isoforms is subcellular localization [18].

Several overexpression and deletion studies have focused on roles of these isoforms in cardiac function. Two independent AC5-deletion (AC5^−/−^) mouse lines have been generated. Overall, deletion of AC5 decreases total cAMP activity in cardiac membranes and isolated myocytes (~35–40%) under basal and stimulated (isoproterenol and forskolin) conditions [19,20]. The two studies reported varying results for changes in cardiac function. Okumura et al. [19] observed a decrease in isoproterenol-stimulated left ventricular (LV) ejection fraction (LVEF) but no alterations in basal cardiac function (with intravenous isoproterenol). Conversely, Tang et al. [20] noted basal changes in the contractile function of perfused isolated hearts in addition to a decreased sensitivity to β_1_-adrenergic receptor agonist. The most notable finding of AC5^−/−^ mice was the effect on parasympathetic regulation of cAMP. Inhibition of cAMP production by Gi-coupled acetylcholine treatment is ablated and Ca^2+^-mediated inhibition is significantly reduced upon AC5 deletion [19]. Physiologically, this corresponds to a reduction in LVEF and heart rate in response to muscarinic agonists and an attenuation of baroreflexes [19,20]. Similarly, AC6 deletion results in a significant reduction of cAMP production in stimulated LV homogenates or cardiac myocytes (60–70%), with no changes to basal cAMP production [21]. AC6 deletion revealed a number of unique contributions not observed in AC5^−/−^ including impaired calcium handling, which results in depressed LV function [21]. In addition, levels of AC6, but not AC5, limit β-adrenergic receptor (βAR) signaling in heart [22,23].

In addition to cardiac contractility, AC5 and AC6 play important roles with regard to cardiac stress. Deletion of AC5 is protective in a number of models of cardiac stress, including transverse aorta constriction, chronic isoproterenol infusion, age-related cardiomyopathy, and high-fat diet, but not overexpression of Gq [19,24,25,26]. While knockout of AC5 can be beneficial to heart, overexpression of AC6 in heart infers protection in response to myocardial ischemia or dilated cardiomyopathy [27,28,29], but not chronic pressure overload using transverse aorta constriction [30]. However, the protection provided upon AC6 overexpression is independent of its catalytic activity as expression of catalytically inactive AC6 is also cardioprotective [31], but requires proper localization via the N-terminus of AC6 [32]. In fact, the expression of AC6 using adenoviral vectors for the treatment of heart disease is currently in clinical trials [33]. Therefore, it is tempting to simplify the system and suggest that AC5 is largely associated with stress responses while AC6 is necessary for calcium handling and contractility. For these reasons, there has been considerable interest in AC5-selective inhibitors for the treatment of heart disease. However, deletion of AC6 can also be protective from chronic pressure overload in female but not male mice [34]; therefore, roles for AC isoforms may depend on the type of heart disease model. AC inhibitors such as Ara-A (Vidarabine) do have benefits for the treatment of myocardial ischemia when delivered after coronary artery reperfusion in mice [35]. However, Ara-A and related AC inhibitors are not selective for AC5 over AC6, although they show considerable selectivity over other AC isoforms [36,37]. Therefore, any benefits of Ara-A likely arise from inhibition of both AC isoforms. However, this could prove risky as AC6 deletion increases mortality during sustained catecholamine stress [38].

**Figure 1:**
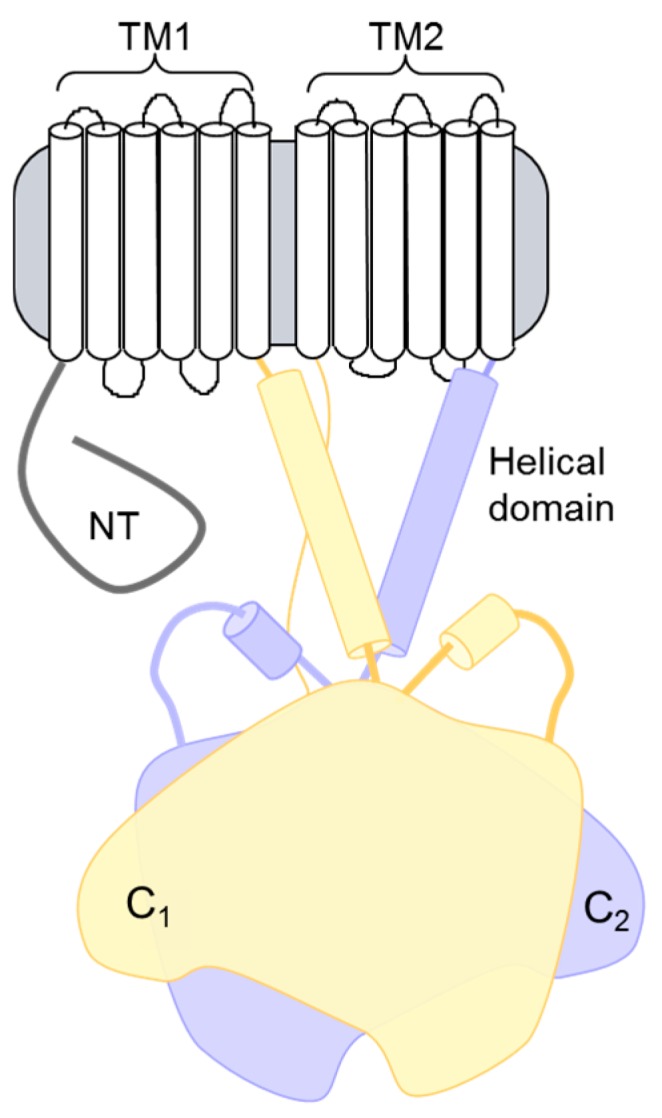
Topology of adenylyl cyclase (AC) isoforms. The structural topology of mammalian membranous ACs consists of an N-terminal (NT) domain followed by a repeating set of transmembrane, helical dimerization, and cytoplasmic domains. The two cytoplasmic domains (C1 and C2) make up the catalytic core and the binding site for many regulatory proteins.

Surprisingly, no polymorphisms that give rise to cardiovascular disease are known to occur in ACs [39]. However, mutations in AC5 are linked to familial dyskinesia with facial myokymia (FDFM), a disease characterized by uncontrolled movement of limb and facial muscles [40,41]. These patients may also have a predisposition to congestive heart failure [41]. Two FDFM mutations occur in a newly appreciated region of AC5, a helical domain that is present immediately after the transmembrane domain and precedes the catalytic cyclase domain (Figure 1). In other nucleotidyl cyclases, this domain forms a tight hairpin to induce an active dimeric conformation of the catalytic domains [42]. Thus, the helical domain may play a role in the stability of the catalytic core or direct regulation of activity.

Roles for additional AC isoforms in cardiac function have been largely overlooked. AC1 was proposed to function as the calcium-stimulated AC in the sinoatrial node that modulates the I(f) pacemaker current [43,44]. However, AC1 knockout mice are not reported to have a heart rate defect and RNA sequencing detects higher expression of AC1 in the right atrium versus the sinoatrial node [45]. Roles for AC2 and/or AC4 are unknown. Currently, a knockout of AC4 is unavailable and AC2 knockout mice display no cardiac phenotype, although RNA for AC2 is elevated in pediatric dilated cardiomyopathy subjects [46]. Cardiac functions for AC9 are discussed below.

## 3. The AKAP Connection: Generating Specificity for AC Function

**Figure 2:**
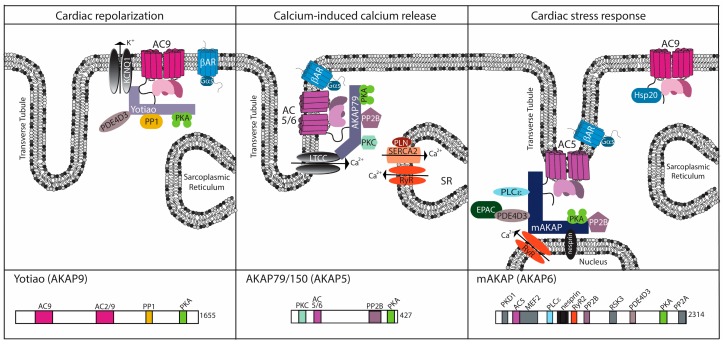
Cardiac AC complexes. AC-associated A-kinase anchoring protein (AKAP) complexes localize to distinct locations within the cardiomyocyte to facilitate physiological function. For each AKAP, a subset of known binding partners and their interaction sites are represented. The model is based upon functional localization of the AC complexes.

Tissue distribution and regulation provide one mode for how the AC isoforms contribute to distinct physiological functions [8,15]. Another mode of signal specificity comes from the formation of AC macromolecular complexes through the scaffolding family of A-kinase anchoring proteins (AKAPs). AKAPs not only facilitate cellular localization of ACs but they also enhance temporal regulation of cAMP signaling. A number of AKAPs exist in heart including AKAP15/18, AKAP79/150, Yotiao, mAKAP, AKAP-Lbc, and Gravin [47] (Figure 2). In the heart, the spatial and temporal regulation by AKAPs provide an important mechanism to facilitate stress response. The associations of ACs with AKAPs facilitate regulation of PKA, downstream effectors, and ACs. This was shown in the dorsal root ganglion where the activation of the transient receptor potential vanilloid 1 (TRPV1) channel by forskolin or prostaglandin E2 is facilitated by AKAP79-AC5-PKA-TRPV1 complex formation and shifts the response to lower concentrations of forskolin by ~100 fold. Disruption of this complex attenuates sensitization of the channel, as anchoring of both PKA and AC5 were required to elicit the maximal effect on TRPV1 current [48]. Anchoring of PKA and ACs to AKAPs can also regulate AC activity. Association of AC5/6 with AKAP79/150 creates a negative feedback loop where cAMP production is inhibited by PKA phosphorylation of AC5/6 [49]. Although this complex feedback mechanism was defined in the nervous system, modulation of TRPV1 in heart is suggested to influence cardiovascular response to disease and injury [50]. Fine tuning of the signal is important for modulating a number of effectors contributing to physiological function.

AC localization is assessed primarily through functional roles of associated complex members enriched at various cardiomyocytes substructures (Figure 2). Association of both AC5 and AC6 with AKAP5 suggests localization at the t-tubule based upon functional association with calcium-induced calcium release [51]. However, with respect to β adrenergic signaling, AC5 is enriched with β2ARin t-tubules whereas AC6 localizes outside the t-tubule [18,52]. Disruption of cAMP compartmentalization is potentially an underlying mechanism of heart failure [52]. AC9 association with Yotiao and KCNQ1 suggests localization at intercalated discs, the sarcolemma, and t-tubules [53]. These AC–AKAP complexes are discussed below.

### 3.1. AKAP5

The AKAP5 family of orthologs are named for their size on SDS-PAGE and for different species—for example, human AKAP79, mouse AKAP150, and bovine AKAP75. AKAP79/150 can associate with AC 2, 3, 5, 6, 8, and 9 as evaluated in tissue culture models [49,54]. In heart, AKAP79 primarily interacts with AC5/6 [51]. The interaction site is located on the N-terminus of AC and in the second and third polybasic domain on AKAP79 (aa 77–153). In cells, AKAP79-scaffolded PKA phosphorylates AC5/6 to inhibit cAMP production [49,54]. This feedback loop allows for precisely timed activation and inactivation of the cAMP signal. Although AKAP79-anchored AC5/6 is inhibited by associated PKA, it is unclear how AKAP79 regulates AC2 activity in isolated plasma membranes.

Physiologically, AKAP79/150 has been studied in isolated cardiomyocytes from wild-type and AKAP150 knockouts. Deletion of AKAP150 significantly reduced stimulated calcium transients and calcium sparks in response to isoproterenol. Additionally, phosphorylation of the ryanodine receptor (RyR) and phospholamban (PLN) was eliminated in cardiomyocytes from knockout mice. It was further shown that AKAP150 forms a complex with AC5/6, PKA, protein phosphatase type 2 (PP2B or calcineurin), Ca_v_1.2, and caveolin 3 (CAV3). This complex is found on t-tubules, while disruption of complex formation upon AKAP150 deletion alters CAV3 and AC6 localization [51]. 

AKAP150 has additional roles that are independent of AC and PKA. AKAP150 localizes protein kinase C (PKC) and L-type calcium channels to the sub-sarcolemma in atrial myocytes enabling regulation of calcium sparklets [55]. TRPV4 sparklets are also modulated by the AKAP150–PKC complex in a distance-dependent manner; a distance less than 200 nM between the TRPV4 and AKAP150–PKC is ideal for proper regulation [56]. AKAP150 is also implicated in β_1_AR recycling. Knockdown or knockout of AKAP150 in isolated myocytes inhibits recycling of β_1_AR back to the membrane after isoproterenol stimulation, but not internalization. Isolated AKAP150^−/−^ cardiomyocytes have an enhanced contraction rate in response to isoproterenol and an increased cell size at basal and stimulated conditions. Based on these results it was postulated that AKAP150 is cardioprotective because the hypertrophy phenotype was enhanced in AKAP150^−/−^ [57].

AKAP150 has been examined in a number of pathology models including myocardial infarction and pressure overload. AKAP150^−/−^ mice were subjected to transverse aortic constriction surgery (TAC) to induce pressure overload; AKAP150 expression significantly decreased in conjunction with a significant increase in hypertrophy, fibrosis, and cell death. Physiologically, deletion of AKAP150 increased left ventricular end diastolic size and impaired fractional shortening after TAC compared to sham animals. Physiological changes were mirrored by alterations in calcium signaling, as AKAP150 creates a complex between the PLN, RyR, and the sarcoplasmic endoplasmic reticulum calcium ATPase 2 (SERCA2). Phosphorylation of RyR and PLN in addition to calcium transients were impaired in response to isoproterenol [58]. A model of myocardial infarction (MI) was also examined in AKAP150^−/−^ mice. Alteration in cardiac signaling that occurs after MI is well documented. MI causes an increase in NFATc3 activation and associated K_v_ channel downregulation. AKAP150^−/−^ cardiomyocytes displayed impaired NFAT translocation in response to phenylephrine, which was dependent on calcineurin activity, preventing downregulation of K_v_ channel currents [59]. Cardiovascular disease is a co-morbidity associated with diabetes [60]. Unlike the other models, in a model of diabetes mellitus, knockdown of AKAP150 ameliorates glucotoxicity-induced diastolic dysfunction in mice. In rat cardiomyocytes from diabetic animals or treated with high glucose, AKAP150 expression is enhanced combined with increased active PKC at the plasma membrane. This, in turn, promotes activation of NFκB and Nox [61], players in the reactive oxygen species pathway that underlie diabetes-induced cardiovascular injury [60]. Thus, while AKAP150 may play a cardioprotective role in some pathology models, this is not always the case.

### 3.2. mAKAP (AKAP6)

Anchored to the nuclear envelope, the cardiac splice variant of muscle AKAP (mAKAPβ) interacts with AC5 to facilitate cardiac signaling [62,63]. mAKAP is localized to the nuclear envelope through its interaction with nesprin [64] while much lower levels of mAKAP are found at the sarcoplasmic reticulum (SR) [65]. While mAKAP is intracellularly located primarily at the nuclear envelope and AC5 is membrane-bound, it is thought that localization of AC5 to the t-tubules allows for this interaction due to the close proximity of the nucleus and t-tubules at sites within the cardiomyocyte [66,67]. AC5 interacts with mAKAP through a unique binding site on the N-terminus (245–340). Similar to AKAP79, PKA binding to the mAKAP complex creates a negative feedback loop to inhibit AC5 activity [63].

A number of molecules implicated in hypertrophy are anchored by mAKAP, including protein phosphatases 2A and 2B (PP2A/2B), PDE4D3, hypoxia-inducible factor 1α (HIF1α), phospholipase Cε (PLCε), myocyte enhancer factor-2 (MEF2) [68], and p90 ribosomal S6 kinase 3 (RSK3) [69,70,71,72,73]. Other proteins are associated indirectly with mAKAP complexes, including EPAC1 and the ERK5 and MEK5 mitogen-activated protein kinases via interactions with PDE4D3 [74]. The interaction of mAKAP and the RyR at the SR promotes phosphorylation and enhances calcium release [75], while RyR located within the nucleus promotes hypertrophy as discussed below.

A role for mAKAP in pathological hypertrophy was first described in mAKAP knockdown myocytes [76] and subsequently shown in mAKAP knockout mice where knockout mice subjected to TAC had reduced hypertrophy, cell death, and did not display TAC-inducible gene expression [77]. Further characterization of the mAKAP macromolecular complex highlights how multiple pathways converge on mAKAP to integrate hypertrophic signaling. The cAMP pathway is integrated through AC5–mAKAP–PDE4D3–EPAC binding to utilize and maintain local cAMP pools [63,69,71,74]. Activated calcineurin is recruited to the complex and is required for nuclear translocation of NFAT [78]. PLCε binds to a complex containing mAKAP, EPAC, protein kinase D (PKD), and RyR2 contributing to PKD activity and nuclear calcium levels [72,79].

### 3.3. Yotiao (AKAP9)

Yotiao is a 250 kDa splice variant of AKAP9 that is present in heart. Yotiao interacts with the alpha subunit (KCNQ1) of the slowly activating delayed rectifier K^+^ current (I_Ks_), a critical component for the late phase repolarization of the cardiac action potential in humans [80]. I_Ks_ is made up of four alpha subunits and accessory beta subunits, KCNE1. Beta-adrenergic control of I_Ks_ by PKA phosphorylation of KCNQ1 increases channel current to shorten the action potential and maintain diastolic intervals in response to an increase in heart rate. Mutations in KCNQ1 are associated with long QT syndrome type 1 (LQT1), a potentially lethal hereditary arrhythmia. Not only do mutations in the KCNQ1 lead to this disease, but mutations within Yotiao (LQT11) and KCNE1 (LQT5) can also give rise to LQT syndrome [81,82]. A subset of these mutations in either Yotiao (S1570L) or KCNQ1 (G589D) disrupts the KCNQ1–Yotiao interaction, resulting in altered regulation of the I_Ks_ channel [81].

Yotiao creates a macromolecular complex between KCNQ1 and important regulators of KCNQ1 phosphorylation. Yotiao scaffolds both positive (PKA) and negative regulators, protein phosphatase 1 (PP1) and phosphodiesterase 4DE3 (PDE4D3), of KCNQ1 phosphorylation [80,83,84,85]. Loss of this scaffold decreases cAMP-dependent PKA phosphorylation of KCNQ1, eliminates the functional response by I_Ks_, and prolongs the action potential [85]. Yotiao is the key to maintaining a tightly regulated feedback loop for I_Ks_-dependent cardiac repolarization and heart rate. Although Yotiao facilitates cardiac repolarization, it cannot overcome channel mutations that alter the capacity for phosphorylation. For example, an A341V mutation in KCNQ1 acts as a dominant negative that reduces basal channel activity and KCNQ1 phosphorylation with no alteration in Yotiao binding [86].

Of the AC isoforms that Yotiao scaffolds (AC 1, 2, 3, and 9) [87], AC9 is the only one present in cardiomyocytes. Unlike the other AKAPs that interact with AC in heart, Yotiao does not scaffold the major cardiac isoforms AC5/6. While Yotiao binds to the N-terminus of AC9, there appear to be multiple sites of interaction of AC9 on Yotiao with the primary site located within the first 808 amino acids and a second, weaker site that overlaps with the AC2 binding site on Yotiao (amino acids 808–956). The interaction of AC9 with Yotiao and KCNQ1 was shown by immunoprecipitation of the complex from cells co-expressing all three proteins, a transgenic mouse line with cardiac expression of KCNQ1–KCNE1, and from guinea pig hearts, which endogenously express the complex. Co-expression of AC9 and Yotiao in CHO cells stably expressing KCNQ1–KCNE1 sensitize PKA phosphorylation of KCNQ1 in response to isoproterenol compared to AC9 or Yotiao expression alone [13]. Yotiao inhibits AC2 and AC3 activity but the mechanism of inhibition is unknown; no inhibition of AC9 activity is observed [87]. Based on these results we would postulate that the AC9–Yotiao–PKA–KCNQ1 macromolecular complex generates a local pool of cAMP that is critical for cardiac repolarization in humans.

## 4. Newly Appreciated ACs in Heart

### 4.1. AC9 Knockout Phenotype

An in-depth look at the role of AC9 in cardiomyocytes has long been overlooked. This is likely due to the low level of expression of AC9 in cardiomyocytes, the fact that AC5/6 accounts for nearly all of the total cAMP production [8], and observations from Antoni showing deletion of AC9 through conventional targeting was embryonically lethal [88]. Interaction of AC9 with the Yotiao–I_Ks_ complex sparked renewed interest in examining its role in cardiac physiology [13]. Meanwhile, the Mutant Mouse Regional Resource Center, an NIH funded strain repository, generated a viable AC9 deletion mouse utilizing a gene trapping cassette. Examination of this AC9 deletion strain resulted in two distinct physiological phenotypes, bradycardia and diastolic dysfunction with preserved ejection fraction; no structural abnormalities were observed in AC9^−/−^ mice using echocardiograms [89]. In addition, Yotiao-anchored AC9 activity is present in the sinoatrial node, supporting a role for AC9 in heart rate. These findings, while intriguing, require further validation. Bradycardia measurements were made while under anesthesia, which has reported effects on heart rate; it will be of interest to see whether the bradycardia phenotype is recapitulated in a conscious mouse model [90]. 

### 4.2. Complexes and Signaling Alterations in AC9^−/−^ Heart

Deletion of either AC5 or AC6, the major cardiac AC isoforms, results in a significant reduction in total cAMP activity, whereas deletion of AC9 is estimated to contribute to less than three percent of total cardiac membrane AC activity (Gαs stimulated). To try and reveal the low AC9 activity in cardiac membranes from WT and AC9^−/−^ hearts, adenylyl cyclase activity was stimulated with Gαs in the presence of the P-site inhibitor, SQ 22,536, which displays >100 fold selectivity for AC5/6 over AC9 [36,89]. This estimate provides only an upper limit to which AC9 contributes to total AC activity in heart. Although the global contribution of AC9 is negligible, it is required for maintaining local cAMP levels in macromolecular complexes. AC9 can interact with two cardiac AKAPs: AKAP79/150 and Yotiao. Yotiao-associated AC activity, as determined by immunoprecipitation-adenylyl cyclase assay [49,63,87], was completely abolished in AC9 knockout hearts, confirming AC9 as the only cardiac isoform associated with Yotiao [87]. Conversely, local cAMP pools associated with AKAP150 were unchanged in AC9^−/−^; AC5 and AC6 are the largest contributors to the AKAP79/150 local pool [51,54].

Similarly, AC9 deletion has a limited impact on global PKA signaling but is important for targeted downstream signaling in local complexes. Although AC9 association with Yotiao sensitizes I_Ks_ phosphorylation in cells [13], it could not be evaluated in the AC9^−/−^ mouse as adult mice do not express a functional I_Ks_ channel [80,89,91,92]. Nonetheless, AC9 deletion reduces basal phosphorylation of the small heat shock protein 20 (Hsp20). Hsp20 and AC9 interact independently of Yotiao as demonstrated by immunoprecipitation and proximity ligation assays. Disruption of this complex by expression of a catalytically inactive AC9 in rat neonatal cardiomyocytes significantly impaired isoproterenol stimulated phosphorylation of Hsp20 [89]. Taken together, this suggests that cardioprotection is yet another role for AC9, by controlling baseline PKA-mediated phosphorylation of Hsp20. Hsp20’s role in cardioprotection is well documented against a variety of insults: prolonged beta-agonist induced hypertrophy, ischemia/reperfusion injury, and doxorubicin cardiotoxicity [93,94,95]. Although AC9 is a binding partner with Hsp20, it is likely that other ACs also bind Hsp20. Deletion of AC9 reduced Hsp20-associated AC activity by only 30%, indicating that other AC(s) interact with Hsp20 in heart [89].

The deletion of AC9 emphasizes the importance of localized cAMP signaling and complex formation, as AC9 contributes to different aspects of cardiac physiology, despite its very low level of activity in heart. AC9 association with the Yotiao–I_Ks_ complex contributes to cardiac repolarization in humans, while an AC9–Hsp20 complex is potentially important for cardioprotection. Further support for an AC9 cardioprotective role in heart comes from an observed upregulation of the micro RNA that regulates AC9 expression (miR-142-3p) in patients with non-ischemic dilated cardiomyopathy and in mouse models of hypertrophic cardiomyopathy [96,97,98,99]. Additional investigations into the role of AC9 regulation of Hsp20 phosphorylation are needed to understand how this complex functions under stress and whether there are other proteins associated with this complex.

### 4.3. AC9 Regulation

Of the AC isoforms, AC9 is the most divergent in sequence and has been the least studied. Expression analysis shows that AC9 is widely expressed in the central nervous system, heart, and other tissues [100,101,102,103]. While the regulatory mechanisms of the other isoforms have been well studied, studies of AC9 regulation have yielded conflicting results. Potential modes of AC9 regulation include stimulation by Gαs, protein kinase C βII (PKCβII) [104], or calcium–calmodulin kinase II (CaMKII) [105] and inhibition by Gαi/o [106], novel PKC isoforms [106], or calcium/calcineurin (CaN) [101]. Determining the regulatory modalities for various AC isoforms is crucial for understanding how the individual isoforms function physiologically. Ideally, the regulation of AC9 would be examined in biochemical and tissue culture models and then confirmed in cardiomyocytes; however, due to the low levels of expression, examining AC9 regulation will prove difficult in this system.

#### 4.3.1. G-Protein Regulation

Every membrane-bound AC isoform is stimulated by Gαs [8]. Compared to AC6, AC9 has a right-shifted Gαs dose-response curve in Sf9 cells, showing a reduced sensitivity to Gαs (TA Baldwin, unpublished observations). This would potentially impact signaling, where a decreased sensitivity to Gαs would reduce downstream signaling outputs, making AC9 even more dependent on complex formation to facilitate local pools of cAMP. Interestingly, all of the alterations in cardiac physiology observed in AC9^−/−^ mice were at basal levels, suggesting that AC9 may be more important for setting the basal tone in cardiac signaling [89]. However, in cells AC9 requires Yotiao anchoring to sensitize the phosphorylation of KCNQ1 in response to isoproterenol [13], emphasizing again the need for complex-dependent signaling.

The original cloning and characterization of human AC9 examined Gαi/o regulation of AC9 in HEK293 cells but did not detect inhibition of AC9 by endogenously expressed somatostatin receptors [101]. Subsequently, Gαi/o regulation of AC9 was reexamined in HEK293 cells upon transient expression of the dopamine receptor (D2L); cells treated with a D2L selective agonist had a significant reduction in AC activity. Thus, the researchers concluded that Gαi/o could inhibit AC9 [106]. It is unclear whether the discrepancy between these two studies is due to the type of Gαi/o-coupled receptor, receptor preference for Gαi versus Gαo, or background activity of endogenously expressed AC6. Interestingly, AC9 does not contain the important residues that are required for Gαi binding and inhibition of AC5 [107]. Further studies are needed to determine whether Gαi is a direct regulator of AC9.

Gβγ is another common regulator of AC activity, inhibiting AC1, AC3, and AC8 or stimulating AC2, AC4, and AC5–7 [8,15]. AC9 regulation by Gβγ had been postulated based on neutrophil chemotaxis studies but never tested in cells [108]. Gβγ does not regulate basal or Gαs-stimulated AC9 activity when assaying membranes from Sf9 cells overexpressing AC9 (TA Baldwin, unpublished observations). Despite not having a direct regulatory role, Gβγ binds the N-terminus of AC9 [89,109].

#### 4.3.2. Kinase and Phosphatase Regulation

Gq regulation of AC9 through CaMKII and PKC was examined in HEK293 cells expressing AC9 stably with transient transfection of either the muscarinic receptor M_5_ or the serotonin receptor 5HT_2A_. Treatment of cells with the receptor agonists (M5, carbachol or 5HT_2A_, 5HT) potentiated AC9 activity in the presence of isoproterenol; expression of the constitutively active Gαq mutant (Q209L) showed similar results. Co-treatment with receptor agonists and the PKC inhibitor, bisindolylmaleimide, further potentiated activity suggesting that PKC acted as an inhibitor of AC9 activity. The authors also examined potentiation of AC9 activity by calcium/calmodulin (CaM) kinase (CaMK) through the M_5_ receptor, by treating cells with carbachol in the presence of a CaM (W-7) or CaMKII (KN-93) inhibitor; both inhibitors reduced AC9 activity. Thus, Gq potentiation of AC activity occurs through activation of CaMKII. However, the authors could not determine whether AC9 is directly phosphorylated by these kinases or whether regulation was via an indirect mechanism [105].

AC9 is also important for neutrophil chemotaxis through activation of AC9 by PKCβII. Neutrophils express high levels of AC9 and have also been used to examine AC9 regulation. Knockdown of AC9 in a neutrophil cell line was shown to inhibit chemotaxis in response to fMLP caused by a decrease in cAMP in extending pseudopods [108]. This mechanism was further dissected to show that PKCβII knockdown recapitulated the AC9 knockdown phenotype. It was proposed that AC9 phosphorylation by PKCβII was the mechanism for increased cAMP in neutrophils leading to chemotaxis [104].

AC9 was originally cloned from a mouse as a calcineurin-inhibited isoform [110]. In HEK293 cells expressing mouse AC9, activity was inhibited by calcium in a concentration-dependent manner but was restored by increasing treatments with the calcineurin inhibitors FK506 or cyclosporin A [100,110]. Subsequent characterizations of human AC9 show conflicting results for calcineurin inhibition [101,102]. The discrepancy is suggested to occur due to differences in variants of AC9 mRNA. Overall, it is unclear whether regulator differences reported for AC9 are due to different expression systems, species differences, or interaction with cell-specific proteins (including AKAPs).

## 5. Conclusions

Multiple distinct AC complexes exist in heart and are important regulators of cardiac physiology. While great strides have been made to understand the composition and roles of these complexes, there are still many questions left to answer. Pharmacological targeting of AC isoforms has been actively pursued but obtaining isoform specificity is difficult, especially for AC5 and AC6. An alternative and widely considered approach is the targeting of specific protein–protein interactions within the cardiac AC complexes. Targeting components of a complex could provide specificity, unlike pan enzyme inhibitors, as these complexes frequently contain only a small percentage of the total protein in the cell. This was the idea behind disrupting the AC5–mAKAP complex as mAKAP-localized cAMP signaling is involved in cardiac hypertrophy [63]. While disruption of this complex was proposed to have a beneficial effect on hypertrophy, the opposite effect was observed. In cardiomyocytes, disruption of AC5–mAKAP binding leads to cellular hypertrophy through an increase in cAMP levels. As previously discussed, AC5 binding to the mAKAP complex creates multiple feedback loops to inhibit cAMP production. These data show how important the fine-tuning of cAMP signaling is and emphasizes the need for extensive studies when designing AKAP complex disruptors for therapeutic use. Finally, many ACs interact with up and or downstream effectors through AKAP-facilitated interactions. However, there is still the possibility that other AC–AKAP complexes have yet to be identified. Currently, an AKAP is not known to interact with the AC9–Hsp20 complex. Moving forward, the possibility of AC complexes independent of AKAPs should also be considered.
